# Rapid review: Ten ways to improve support for minoritised informal adult carers at local government policy level to redress inequality

**DOI:** 10.1016/j.puhip.2024.100543

**Published:** 2024-08-26

**Authors:** A. Barnes, F. Phillips, K. Pickett, A.J. Haider, J. Robinson-Joyce, S. Ahmed

**Affiliations:** a Public Health and Society, Health Sciences, University of York, UK; b Bradford Health Determinants Research Collaboration (HDRC), City of Bradford Metropolitan District Council, UK; c NHS Bradford District and Craven, UK; d Commissioning, City of Bradford Metropolitan District Council, UK

**Keywords:** Unpaid carers, Informal caregiving, Minoritised groups, Support, Local policy, Rapid review, Inequality

## Abstract

**Objective:**

To rapidly identify and summarise evidence on key factors that affect access to support for minoritised informal adult carers which could be addressed at the level of local government policy-making.

**Study design:**

Rapid evidence review.

**Methods:**

A rapid umbrella review was undertaken of systematic reviews of qualitative, quantitative and/or mixed method studies. Systematic reviews were identified through database searches (Medline, Cochrane, Proquest), key author searching, referrals by experts (n = 2) of key reviews, and citation and reference checking of identified reviews in September–October 2023. Systematic review evidence was supplemented with grey literature identified by practitioners (n = 2) as locally-relevant. Data was extracted directly into a table and findings synthesised narratively by theme.

**Results:**

Many factors were identified as affecting access to support for minoritised unpaid adult carers, including: inattention to socio-cultural diversity; issues of representation, racism and discrimination; and socio-economic inequality. Factors were themed around ten areas for local action, including: the importance of recognising intersectional disadvantage and diversity; ensuring support is socio-culturally appropriate; paying attention to gendered hierarchies in service design; identifying and ‘designing out’ racism and discrimination; addressing exclusions that minoritised carers with additional communication needs face; mitigating socio-economic inequality; and taking a ‘whole system’ approach that improves integration, routine data collection and support service evaluation.

**Conclusions:**

We identified ten potential ways in which inequalities in support for minoritised unpaid adult carers could be addressed locally. Although the existing evidence base is limited, these ten areas could usefully be targeted for further investigation in research and within local policy development.

## 1 Introduction

Informal caregivers have an essential and growing role in the UK and Europe, looking after an increasing number of people in need of long-term care [1,2]. While definitions of informal care vary across research and policy contexts [3], an informal carer can be understood as someone who helps look after a family member, neighbour or friend without being formally paid; for example, providing personal care; monitoring medications of those with a chronic illness, disability, or other long-term condition; and doing practical tasks, including shopping, collecting prescriptions, and helping with bills and finances [4].

Informal carers are recognised to have a critical role within health and care systems [5]. Yet it is also recognised that taking on a caring role, particularly if it involves long hours of care, has significant impacts on people's lives, health and wellbeing, often resulting in poorer physical and mental health, and quality of life [1,5,6]. It is therefore essential from a public health perspective that carers receive quality, consistent support [5].

Supporting carers can take different forms, including wellbeing or advocacy services that are directly targeted towards carers, workplace support, and peer or lay support via social networks within communities; and can involve practical (including financial), physical or psycho-social assistance [1,5]. While the importance of quality support is established and there is policy interest in achieving this across the UK and Europe, little is known about factors that shape access to such support for unpaid adult carers at a local level, and particularly for minoritised carers, defined here as:

“*individuals and populations (including numerical majorities) whose collective cultural, economic, political and social power [access to resources and health] has been eroded through the targeting of identity [including based on ethnicity, language, religion, age, sexuality, migration status, disability, neurodiversity, socio-economic background)] in active social processes that sustain [existing systems of dominance]”* [7].

Carers in minoritised groups, particularly minoritised ethnicities, are increasing in the UK and Europe yet often do not access formal support services [8]. There is some evidence to suggest that carers in minoritised ethnicities, including Black, Asian and other minoritised ethnic groups, and LGBTQ + carers are less satisfied with social care and support services, and have poorer health outcomes [8,9]. The implications of inequalities in support are also suggested in recent UK research highlighting how identifying as a woman, of an ethnic minority or in a lower socio-economic group interacts with caring, resulting in greater negative effects in key life domains [6]. While recent papers highlight that minoritisation, as a social and political process, provides a “context for caregiving”, intersecting with personal, relational, situational and socio-cultural factors to produce differing experiences, coping strategies, levels of distress, and support needs and access [10,11] there is limited research on these intersections [11].

It is in this context that local policymakers are seeking to develop better support systems for informal carers. In Bradford, for example, a new district strategy for unpaid carers was being developed in 2024 but it became clear during the strategy development process that there was a lack of clear information available to decision-makers about key factors shaping support for unpaid adult carers in minoritised groups, which could be addressed locally. Given this gap, the University of York/Bradford Council Health Determinants Research Collaboration (HDRC) Policy Hub completed an independent rapid review on this topic to inform local policy development. The aim was to rapidly summarise evidence on factors that affect inequalities in access to support for unpaid adult carers in minoritised groups, synthesising this for relevance at local policy level. The Bradford HDRC, funded by the National Institute of Health Research (NIHR) has a remit to support evidence-based decision making in a local authority setting. This paper presents the methods and results of the rapid review.

## 2 Method

A rapid umbrella review of systematic reviews was completed within limited time (two months), resources and budget to provide insights to local policy makers in Bradford. Rapid reviews rationalise systematic review methods in order to balance academic rigour with meeting the needs of practice: getting evidence to policymakers quickly [12]. Standardised rapid review methods do not exist, particularly for those not primarily focused on intervention effectiveness or cost-effectiveness, and because methods need to be adapted to meet the needs of practice. Relevant Cochrane [12] and Health Policy and Systems guidance [13] on rapid reviews was therefore modified for applicability and used to structure this review.

### 2.1 Searches

Searches were carried out with a view to identifying ‘key’ review articles of relevance for, as well as evidence sources that practitioners identified as important to consider in local policy development (rather than comprehensively identifying all review articles, as would be the case in a systematic review). Systematic reviews were identified through simple database searches (Medline, Cochrane, Proquest) using broad and simple search strings (e.g. "unpaid carer" OR "informal care*" AND minorit*; carer AND ethnic*) and systematic/literature review filter in September–October 2023, as well as through referral of key reviews by experts in the field (n = 2). Reference checking and citation searching of all included reviews was completed. Key author searching was also completed (e.g. Greenwood). Evidence from systematic reviews was supplemented with literature identified by local practitioner experts (n = 2) as relevant to local policy development.

### 2.2 Inclusion criteria

The RR included: systematic literature reviews of any type and literature identified by local practitioner experts as relevant to local policy development which provided insight into factors affecting inequalities in support for unpaid adult carers from minoritised groups (e.g. marginalised or discriminated against due to targeting of ethnicity, sexuality, neurodiversity, disability or other actual or perceived aspects of identity), with or without information about implications for, or action that could be taken in, local policy or practice; written in English; of relevance to England (i.e. included material on UK, European or other high-income contexts); and published from 2000.

### 2.3 Screening and selection of reviews

Titles and abstracts were screened by one academic reviewer against inclusion criteria. There was insufficient resource for second checking as is common in RRs [13]. Two-stage screening was used, with initial flagging of possible sources for inclusion to identify those for full document review. Evidence excluded at full document review with reasons for exclusion were recorded.

### 2.4 Data extraction and synthesis

To rapidly complete the review, key characteristics of included evidence (date, dimensions of marginalisation/discrimination that the source focused on (e.g. ethnicity, gender identity), context of caring (e.g. dementia, palliative care)) and a brief summary of findings relating to factors shaping inequalities experienced by minoritised adult carers, and issues amenable for local action (with any identified examples) were extracted directly into a table. These factors and issues were subsequently themed around key areas for local action, then summarised and synthesised narratively by theme. See Supplementary File 1 for an example of our final data extraction table and Supplementary File 2 for a note about language used in the synthesis. The full data extraction table is available on request from authors.

### 2.5 Evidence quality

The RR gives an overview of factors affecting support for minoritised carers as reported within key existing reviews and selected grey literature identified by practitioners as important in local policy development. We used the JBI systematic review checklist for review articles and the AACODS checklist for grey literature to consider quality of identified literature (see Supplementary Files 3 and 4). Only a small proportion of reported data was relevant in some systematic reviews, with a lack of detailed review evidence overall on the topic: only six review articles were rated as of high quality and relevance to the rapid review (see sources highlighted in yellow in Supplementary File 4). We also included grey literature, which was sometimes unclear about the underpinning evidence. We include general reflection on evidence quality within our discussion as it also covers limitations and indicate in the synthesis any concerns or where reported findings may be of limited relevance.

## 3 Results

**Fig. 1:**
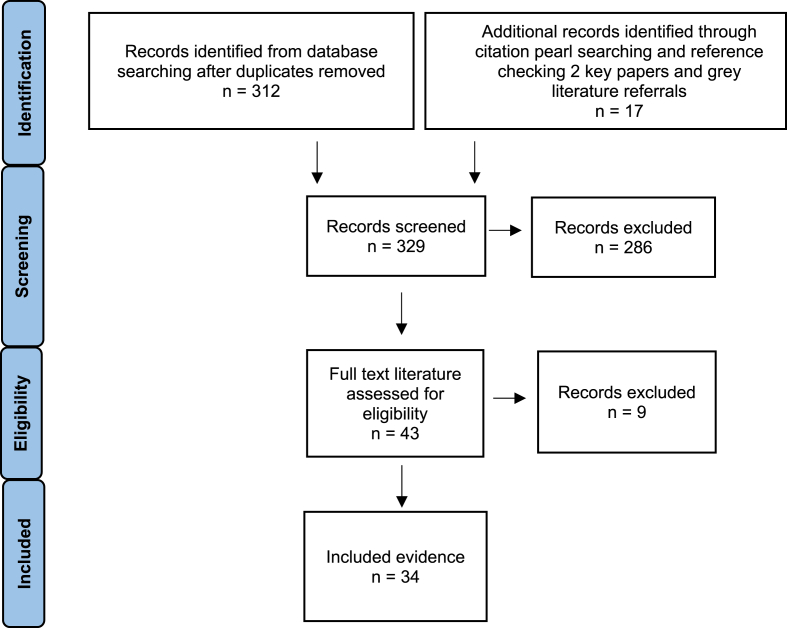
PRISMA diagram indicating process of rapid literature search and selection.

A total of 34 sources were included: 27 published systematic reviews and 7 grey literature sources referred by local practitioners (Fig. 1). Of the included evidence, 14 sources focused specifically on carers by minoritised ethnicity, 3 by sexuality- or gender-identity, 2 on intersections in minority status, 3 on migration history, and 12 did not focus on a specific minoritised population. Twelve sources focused specifically on caregiving in relation to dementia, 2 in relation to cancer, 1 to stroke, 1 to mental health, 2 to palliative care, with 16 sources focusing on carers more generally (see Supplementary File 4 for summary characteristics of all included sources).

### 3.1 Synthesis of findings

A range of factors were identified as shaping inequalities in support for unpaid adult carers from minoritised groups, including: issues of representation, racism and discrimination; inattention to socio-cultural diversity; and socio-economic inequality. Factors were themed around ten areas for local action as presented below.

#### 3.1.1 Understand and recognise diversity within minoritised groups

Included evidence highlighted the diverse identities of minoritised unpaid adult carers (e.g. by ethnicity, language, religion, age, gender, sexuality, migration status, disability, neurodiversity, socio-economic background) and risks of exclusion when there is inattention to such diversity and experiences of racism and discrimination within systems of support [11,[16], [17], [18]] Understanding the concept of intersectionality was suggested as potentially useful in one source: differing identities of individuals and populations intersect in ways that affect how they are viewed, understood, and treated ‘by others’ in society, including within health and social care systems, meaning that some people face multiple, accumulating experiences of discrimination, marginalisation and disadvantage [11]. To address intersecting inequalities, included studies suggested that local policy needs to understand, recognise and value diversity; commit to changing the status quo of service planning and delivery; and proactively identify and engage with unpaid adult carers in minoritised groups, to identify and redress barriers, marginalisation and unequal influence within health and care systems [16,17,19,20].

#### 3.1.2 Ensure support is socio-culturally appropriate

Included studies highlighted how limitations in health and care systems around the delivery of socio-culturally-acceptable support (e.g. heteronormative assumptions, ethnocentrism) can exclude adult caregivers in minoritised groups, including LGBTQ+, Black, Asian and other ethnically minoritised carers, leading them to withdraw from, or not use, care and support services [9,18,21,22] There was a need highlighted for better socio-cultural support, but included evidence was not clear on what this meant in practice. It was reported that there was lack of understanding about the preferences and satisfaction of ethnically minoritised carers with existing support [8]. Included sources reported that minoritised carers (including of Black, Asian Gypsy, Roma and Traveller backgrounds, carers who are refugees or seeking asylum, LGBTQ + carers, carers with a disability, and carers with autism) are more likely to feel services do not meet their needs, have concerns about them and/or experience difficulties in accessing support [9,16,17,23].

Included sources also highlighted that support services need to be sensitive and adaptive to different socio-cultural needs (e.g. different languages), preferences (e.g. due to social norms) and past experiences, including of discrimination to address inequality, and investigate how existing support may actively disadvantage carers in hidden and unintended ways [8,19,[22], [23], [24], [25]]. One review focusing on ‘culturally and linguistically diverse’ older adult informal caregivers found that while, for some people, culturally and linguistically ‘matched’ providers were preferred, the receipt of support from skilled, respectful, and culturally-sensitive providers who were not of the same culture/language was acceptable to others [19].

It was reported that delivering adaptive, socio-culturally appropriate support can be challenging due to inadequate staff knowledge or competence, and services finding it difficult to involve minoritised groups [8,16,17,21]. Yet coproduction of support strategies and services was noted as important for: rectifying deficits in power and influence, supporting learning, and ensuring different needs are understood and met [16,17,19]. Included sources suggested that a ‘cultural humility’ learning framework within support services, focusing on not making assumptions about people's background, identity, or needs; critical self-reflection; and recognition of power imbalances, may promote respect and optimise support for all [16,17,19]. Other examples of inclusive local support activities identified in included evidence were: using diverse language and imagery and marking different socio-cultural and/or religious events (e.g. Eid, Diwali, Race Equality Week, Black History Month, LGBTQ + events), with the latter providing opportunities for shared learning, particularly if co-developed with minoritised carers to ensure inclusion [16,17,19].

#### 3.1.3 Recognise different understandings of care and carer coping strategies within local policy development and support service design

Included evidence reported that different cultural understandings of care (e.g. given that ‘carer’ does not translate in some languages including Bengali, Gujarati, Urdu, Punjabi) and expectations about “familial duty” mean that some carers, including carers in some minoritised ethnicities, may primarily see themselves as a family member or friend, shaping whether, how and when support is accessed [3,8,17,[22], [23], [24],^26^]. It was also highlighted that, if parental caring is an expected duty, there is a risk caregivers feel their role is unappreciated by family and friends, and embarrassment or shame about accessing support, taking a break, or seeking employment [17]. It was noted that socio-cultural norms also affect caregiving coping strategies, which need to be taken into account when providing support. For example, religious strategies can be important within some minoritised ethnicities [3,22,26,27] and, among some families with migration histories to Europe. One review highlighted new coping approaches developing: while older relatives who migrated may prefer co-residence with family, staying nearby, rotational caregiving or visiting older adults are emerging [26].

Included sources indicated that socio-cultural norms also affect understandings of the condition of the person being cared for, shaping how support needs to be provided. For example, it was in noted in three reviews and one practice report that in most South Asian languages, there is no word for ‘dementia’ and research finds lower dementia awareness in some communities (e.g. Chinese diaspora) [17,[23], [24], [25]]. It was reported that some carers may therefore need support to recognise, ask for help in relation to, and/or combat any stigma around dementia or other health conditions (e.g. mental health) [24,28], including through the use of culturally-appropriate training and language: one review indicated, for example, that older people from some ethnic minority groups may value the opportunity to talk about depression when described as isolation, loss or bereavement [29].

Two practice reports indicated that targeted outreach to reach minoritised carers (e.g. working with key community figures, leveraging relationships of local voluntary or faith organisations, including language providers to reach refugees and people seeking sanctuary) may be useful to enable recognition as a ‘carer’ and access to support [8,16,17] In England, it was suggested that such outreach can be a means for identifying carers who would otherwise not have considered a ‘Carers Assessment’: ‘assessment’ can be perceived as a judgement of caregiving and particularly stigmatising for LGBTQ+ and/or disabled carers [16].

#### 3.1.4 Ensure gendered hierarchies and inequalities in care work are considered when developing support

Included sources indicated that family structures, expectations, and gendered hierarchies often mean women become caregivers, across cultures and ethnicities, but that this can particularly be the case for women in some racially and ethnically minoritised groups, including in the context of intergenerational caregiving within South Asian communities [11,22,24,26]. One systematic review reported that among families with migration histories to Europe, family members, friends, and neighbours may provide as much as 90 % of in-home long-term care to older adults, with caregiving responsibilities remaining largely (though not exclusively) with women family members [26]. Another review highlighted however, that gendered caregiving responsibilities can change with age over the lifecourse [11]. It was indicated that gendered responsibilities, hierarchies and inequalities in care work need to be considered when developing support [26].

#### 3.1.5 Identify racism and discrimination and ‘design it out’ of services

Included sources highlighted that racism and discrimination exacerbate the challenge of caregiving [8], with inequalities in access to, experiences of, and outcomes of support services rooted in experiences of racism, discrimination and/or marginalisation [8,20,23]. A number of systematic reviews and practice reports indicated that caregivers in minoritised ethnicities, and those they care for, report experiencing racism and disrespect (e.g. use of cultural stereotypes, preconceptions) in health and social care systems, leading to distrust, frustration, cessation of support service use and feelings of neglect and exclusion [17,19,23,25,26]. Past experiences of racism and discrimination can also reduce the likelihood of ever seeking support in the first place [23]. It was reported that discrimination, ethnocentrism and service gatekeeping, and mistrust of providers have, for example, been reported in relation to dementia and palliative care, affecting support access and preferences among Black and other minoritised ethnic groups [[30], [31], [32]].

Similarly, one practice report and a systematic review indicated that harassment, prejudice and structural discrimination (including past legal discrimination) faced by LGBTQ + carers and heteronormative assumptions within services about sexuality and relationships, may lead LGBTQ + carers to distrust and be reluctant to access support [17,33]. There is practice-based evidence to suggest that some carers report continually having to ‘come out’ to services, which can be distressing [17], and that the autistic needs of adult carers also tend to be neglected, with reasonable adjustments not made locally, leading to isolation [34].

Included practice reports suggested that creating safe spaces for staff and carers to have open conversations about race, ethnicity, sexuality, and other aspects of identity may build trust and address past negative experiences [16,17]. Included reviews suggested that staff training in anti-racist and/or anti-discrimination pedagogy may help ensure that support meets needs [19] and that adult carers disclosing racism and/or discrimination should be referred to advocacy and rights-based support [28].

#### 3.1.6 Ensure access to social networks for support, influence and voice

Included evidence highlighted that strong social networks provide adult carers with emotional and practical assistance [5]: isolated carers can struggle to find needed help and are at greater risk of poor mental and physical health. It was therefore indicated that it is important that all carers can access support from their wider socio-cultural communities [21,22]. Social networks can be particularly important for carers in minoritised groups, given risks of societal marginalisation, exclusion and discrimination. UK practice-focused reports highlighted, for example, how difficult or estranged family relationships, when families are not accepting of LGBTQ + identity, can isolate LGBTQ + carers, with implications for belonging and self-esteem [16]. Strong relational or cultural ties within some minoritised groups can be undermined by caregiving itself: one review highlighted how mental health can be compromised among people of South Asian backgrounds who have experienced stroke and their carers, due to isolation from South Asian community members [22].

Practice evidence included in the rapid review highlighted the importance of giving carers information on peer support opportunities locally - to share experiences, exert power and voice, receive emotional support and practical advice [5] Practice reports noted that Black, Asian and ethnic minority support networks may be helpful for unpaid adult carers from minoritised ethnicities [17] and that, while some LGBTQ + carers might appreciate LGBTQ + specific groups [33], others might prefer to focus on their caring role: speaking to carers about what they need is important, rather than making assumptions [16]. It was noted that holding support groups in trusted spaces (e.g. religious buildings, community centres) or co-hosting events with local cultural forums may promote involvement [17].

#### 3.1.7 Address exclusions that adult carers with additional communication needs face

**Fig. 2:**
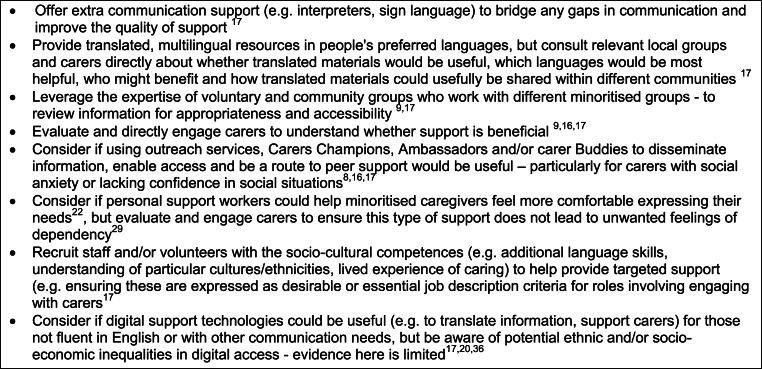
Examples of local policy actions to redress exclusions that adult carers with additional communication needs face.

Two practice reports and a systematic review highlighted that adult carers with additional communication needs (e.g. physical, sensory or learning disabilities, people who do not speak or read English fluently – including some recent migrants) may experience exclusions from support if designed around dominant ways of communicating [17,22,29]. It was noted that written and spoken levels of English fluency can vary across different groups, including, for example, older people with histories of migration to Europe, with lower levels reported amongst some older people of Pakistani, Bangladeshi, Chinese, Vietnamese, and Somali backgrounds in the UK [29]. One review indicated that significant challenges have been reported for recent migrant cancer caregivers with limited language proficiency in high-income settings [35], and another reported concerns raised about providers offering lower care quality and respect to people not fluent in English [22]. Fig. 2 details examples of local policy actions that could be taken, extracted and summarised from across a number of the included sources.

#### 3.1.8 Identify and mitigate socio-economic inequalities and support livelihoods

It was reported in a limited number of included sources that minoritised adult carers often face intersecting socio-economic inequalities that affect access to support: financial resources are a critical factor affecting the support unpaid carers receive [9]. Many carers across all social groups find they have higher bills, need to reduce working hours or quit jobs, with income loss causing significant financial strain [5]. Included practice-evidence from the UK highlighted how the cost-of-living crisis was putting pressure on carers, with a quarter cutting back on essentials [9]. Practice-reports also highlighted that carers from Black, Asian and minority ethnic backgrounds were more anxious about their financial situation and more impacted by local service closures than White carers in the UK [9,17].

The need for financial, housing, mental health and/or access to employment support for ethnic minority carers was highlighted in a few of the included studies, to mitigate adverse material circumstances linked to societal marginalisation and discrimination [28]. Supporting livelihoods by helping carers stay in or return to work or access social security was reported as helpful, particularly for minoritised women who often do most care work [9]. It was noted that many carers do not claim the social security benefits to which they are entitled [5]. Local action to address financial inequality (e.g. targeted financial, benefits advice) may help alleviate financial caregiving burdens: one review highlighted, for example, the particular importance of advocacy and advice for caregivers to people recovering from mental health conditions [28].

Work commitments were noted in included sources to be a barrier to accessing support. A recent practice-report highlighted that LGBT + carers are more likely to say work commitments are a barrier to accessing services [16]. Yet for trans people, there is evidence to suggest that accessing employment can be difficult due to discrimination, with particular implications for financial security, mental health and wellbeing [16]. Two reviews suggested that carer-friendly workplace policies can support livelihoods [37,38]. It was noted that Black, Asian and minority ethnic carers are less likely to be retired and under the age of 65 [9], and so workplace action may be particularly important for such carers. Two reviews highlighted that there is limited research however, to understand how workplace policies can be optimised for all [37,38].Flexibility to enable carers to juggle work and care; access to paid carer's leave; and explicit recognition of carer skills in recruitment and promotion were all examples of local workplace support highlighted in included sources [9,37].

#### 3.1.9 Ensure representation of minoritised unpaid adult carers in systems of support

A limited number of included sources highlighted that lack of diverse representation of minoritised groups in support organisations, healthcare and policymaking can affect the sufficiency and appropriateness of support [9,21]. It was suggested that minoritised carers may feel they have ‘no voice’ locally and unable to raise service concerns [17] and that ensuring diverse representation in decision-making may lead to more inclusive and culturally-sensitive support initiatives [9,17].

#### 3.1.10 Ensure there is a ‘whole system approach’ to support carers, including attention to improving data systems and evaluation

It was reported that minoritised adult carers are comparatively more likely to have poor physical and mental health than other population groups [5,8] and carers who are unable to address their wellbeing needs find it increasingly challenging to caregive effectively, with knock on effects for those they care for, local service demand and prescribing costs [5]. Given this, included sources indicated that a whole system approach, involving collaboration and integration across health and social care was essential to support carer wellbeing and redress deficits in influence, racism, discrimination, and marginalisation [9,17], with multidisciplinary input from across government, the voluntary sector and health and social care important here [5]: to identify gaps and develop comprehensive solutions, including identifying and ‘designing out’ inequalities in access, experience and outcomes [20].

It was noted how integrated support can improve care planning, improve the care (including palliative care) of people with long term conditions, and reduce unnecessary hospital admissions [5]. A key area for integrated action in England highlighted in two practice reports was Carers Assessments [5]: evidence suggests that Black, Asian and other minoritised ethnic groups, and lesbian, gay and bisexual carers, are less likely to have had an assessment compared to White carers and heterosexual carers respectively [9].

**Fig. 3:**
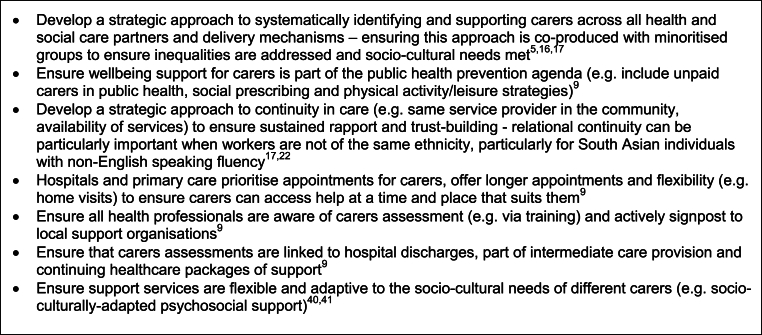
Example elements of an integrated local approach for supporting minoritised unpaid adult carers.

Another important area for integrated local action highlighted in included sources was data systems and evaluation. It was noted that: limited routine data was collected and evidence published about the support experiences and outcomes of different adult carers [8,10,16,17,36]; and that better data systems, including disaggregated demographic and diversity data on service participation and improved data sharing between local carer support organisations is needed [16,17,34]. One included practice report from the UK indicated that outcome data is collected in some local settings using standardised wellbeing scales [34]. It was unclear however, if these had been validated in local practice with considerations of socio-cultural appropriateness: carers of different ethnicities may, for example, interpret and respond differently to questions [8]. Included sources noted that more process and impact evaluations of support are needed locally, exploring what worked well or less well, why and for whom, focusing on different outcomes of interest to different unpaid adult carers and taking into account culture, ethnicity, and structural inequality [17,[39], [40], [41]]. Complexity-focused evaluations allowing for larger samples, including theories of change, mixed methods approaches, and directly involving carers in the evaluative process (e.g. participatory research, participatory video) were noted as being needed [28]. Other example elements of an integrated local approach highlighted in included sources are illustrated in Fig. 3.

## 4 Discussion

This review identified many factors affecting access to support for minoritised unpaid adult carers: inattention to socio-cultural diversity; issues of representation, racism and discrimination; and socio-economic inequality. It also identifies 10 potential ways in which inequalities in support could be addressed locally, including: the importance of recognising intersectional disadvantage and diversity; ensuring support is socio-culturally appropriate; paying attention to gendered hierarchies in service design; identifying and ‘designing out’ racism and discrimination; addressing exclusions that minoritised carers with additional communication needs face; mitigating socio-economic inequality; and taking a ‘whole system’ approach that improves integration, routine data collection and support service evaluation. These areas of action are potentially relevant to local government, health and social care organisations, and voluntary sector groups.

While the review identifies these ten areas for local action, doing so was challenging given the sparsity of rich and detailed review evidence on this topic, which is a limitation of this rapid review. As indicated above, only a small proportion of reported data was relevant in some of the included systematic reviews. More detailed insight was provided about factors shaping inequalities in access to support in the included grey literature, but these were more unclear about the methods or evidence underpinning them. Most of the included systematic reviews provided insight about carers minoritised by ethnicity and caregiving in relation to dementia, with comparatively less insight, for example, about caregivers for other long-term conditions or minoritised sexualities or gender identities; with none of the included papers specifically considering disability or neurodiversity. At the same time, and in line with other studies in this field [11], there was only limited insight in the included evidence about how minoritisation intersects with other factors to shape experiences, coping, distress, needs and access to support [10,11]. In support of other research [[41], [42]], we suggest that more research is needed to better understand factors shaping support for minoritised adult carers and that the ten areas for local action identified here could particularly be targeted for further investigation in local government policy development. A more comprehensive systematic review on this topic could usefully be carried out to address limits to the rapid review approach used in this study, to further test out and explore the 10 important areas for local action identified.

We used the term ‘minoritised’ in this rapid review to reflect our understanding of minoritisation as a relational and political process that creates a disadvantageous context for caregiving [10,11]. We found however, that there was a lack of critical consideration in the included evidence about what ‘minority status’ means or the social categories used. In some cases, the terms ‘minority’ or ‘ethnic minority’ seemed to be applied as descriptive labels [11], rather than constructs representing theorised understandings of the social processes and power relations that affect the everyday context for caregiving and access to support. Not only is this another limitation of the rapid review, but it is also potentially problematic because, without critical consideration of this, there is a risk that issues with access to support become misrecognised as technical service ‘barriers’ to be overcome, rather than more fundamental social and political obstacles embedded within health and care systems and society [43]. Here, there is a potential role for qualitative research, carried out with and alongside minoritised carers, using methods that place carers in a position of power to tell their own stories, and which take account of issues of influence, culture and structural disadvantage [17,39,44]. We suggest that further locally-situated research of this kind is crucial to inform local policy development, for it is only by shifting socio-political conditions that inequalities in support for informal adult carers will be addressed long-term.

## Ethical approval

Ethical approval was not needed as the rapid review did not involve human participants.

## Funding

Barnes and Pickett are funded by the NIHR Yorkshire and Humber Applied Research Collaborations [reference NIHR200166]) and UK Prevention Research Partnership Collaboration (MRC) - ActEarly [reference MR/S037527/1]. The NIHR Health Determinants Research Collaboration Bradford is funded by the PHR Programme (NIHR151305). Content and views expressed in this briefing are those of the author(s) and not necessarily those of the National Institute for Health Research, the Department of Health and Social Care, the UK Prevention Research Partnership/MRC.

## Declaration of interests

Barnes is an academic lead of the University of York/Bradford Council Health Determinants Research Collaboration (HDRC) Policy Hub and was involved in helping to scope up the protocol for this evidence review, carried out the review in full and prepared the manuscript.
Pickett is an academic lead of the University of York/Bradford Council Health Determinants Research Collaboration (HDRC) Policy Hub and was involved in scoping up the local policy and practice need for this evidence review and provided feedback on the findings/manuscript.
Robinson-Joyce and Ahmed work for City of Bradford Metropolitan District Council and were involved in identifying the local policy and practice need for this evidence review and provided feedback on the findings/manuscript.
Haider works for the NHS in Bradford District and Craven, leading on commissioning and procuring support for informal unpaid carers and was involved in identifying the local policy and practice need for this evidence review and provided feedback on the findings/manuscript.
Phillips is the Bradford Council lead for the University of York/Bradford Council Health Determinants Research Collaboration (HDRC) Policy Hub was involved scoping up the local policy and practice need for this evidence review and provided feedback on the findings/manuscript.

## Declaration of competing interest

A. Barnes is an academic lead of the University of York/Health Determinants Research Collaboration (HDRC) Policy Hub and was involved in helping to scope up the protocol for this evidence review, carried out the review in full and prepared the manuscript.

K. Pickett is an academic lead of the University of York/Health Determinants Research Collaboration (HDRC) Policy Hub and was involved in scoping up the local policy and practice need for this evidence review and provided feedback on the findings/manuscript.

J. Robinson-Joyce and S. Ahmed work for Bradford Metropolitan District Council and were involved identifying the local policy and practice need for this evidence review and provided feedback on the findings/manuscript.

A.J. Haider works for the NHS in Bradford, leading on commissioning and procuring support for informal unpaid carers and was involved in identifying the local policy and practice need for this evidence review and provided feedback on the findings/manuscript.

F. Phillips is the Bradford Council lead for the University of York/Health Determinants Research Collaboration (HDRC) Policy Hub was involved scoping up the local policy and practice need for this evidence review and provided feedback on the findings/manuscript.
