# Supplementary material for: Rapid review: Ten ways to improve support for minoritised informal adult carers at local government policy level to redress inequality
Source: Public Health Pract (Oxf). 2024 Aug 26;8:100543. doi: 10.1016/j.puhip.2024.100543 (PMC11405819; doi:10.1016/j.puhip.2024.100543)
Supplement: Multimedia component 2 [file mmc2.docx]

A note about language

This paper adopts the NHS Race and Health Observatory ‘s approach with regards to language: recognising that “language has power” and that the terminology we use when talking about different people and groups can have real world impact. Following the Observatory’s principles, we have tried to be as specific as possible when discussing different people and minoritised groups, only using collective terminology when there has been a legitimate need to do so. Where collective terminology is needed and/or the context is unclear, which was the case in many of the included sources, collective terms such as ‘minoritised ethnic groups’ ‘Black, Asian and other minoritised ethnic groups’ are used. Furthermore, because the rapid review synthesises wider research which does not use terms that reflect our preferred approach, there may be occasions where other terminology is used. The acronyms or initialisms BME or BAME are not used. The collective term LGBTQ+ is used, reflecting its use in the literature.
