# Supplementary material for: Rapid review: Ten ways to improve support for minoritised informal adult carers at local government policy level to redress inequality
Source: Public Health Pract (Oxf). 2024 Aug 26;8:100543. doi: 10.1016/j.puhip.2024.100543 (PMC11405819; doi:10.1016/j.puhip.2024.100543)
Supplement: Multimedia component 3 [file mmc3.docx]

**Appendix 2 - Quality appraisal**

For critical appraisal, we used checklists from the Critical Appraisal Skills Programme (Critical Appraisal Skills Programme: <https://casp-uk.net/casp-tools-checklists/>) relevant to each empirical study design, and the AACODS checklist for grey literature, to consider the quality of identified literature. Appraisal was not applicable for predominantly descriptive or theoretical studies.

**Grey literature - AACODS checklist [modified]**

**0-5 rating 0=low/poor 5= high/good**

| **First author & date** | **Authority** | **Accuracy** | **Coverage** | **Objectivity** | **Date** | **Significance**  **(to specific research area that is a focus of the review)** | **TOTAL** |
| --- | --- | --- | --- | --- | --- | --- | --- |
| Carers UK 2022 | 5 | 3 | 4 | 4 | 5 | Clear focus on unpaid with aspects of inequalities for minoritised groups focused on | 21 |
| Carers UK Carers Assessments.. | 5 | 4 | 4 | 3 | 5 | Clear focus on unpaid with aspects of inequalities for minoritised groups focused on in relation to carers assessments | 21 |
| Carers UK 2023 – Supporting Black… | 5 | 3 | 4 | 3 | 5 | Clear focus on unpaid carers in minoritised ethnic groups | 20 |
| Carers UK Supporting LGBTQ+.. | 5 | 3 | 4 | 3 | 5 | Clear focus on unpaid carers in minoritised groups in relation to sexual/gender identity | 20 |
| Kapadia et al 2022 | 5 | 5 | 5 | 5 | 5 | Only limited aspects of relevance for unpaid carers | 25 – with limits re: significance |
| Moriarty 2008 | 5 | 3 | 3 | 3 | 5 | Some aspects of relevance to unpaid carers | 19 |
| NICE, 2020 | 5 | 5 | 5 | 5 | 5 | Clear focus on unpaid carers -less focus on minoritised groups | 25– with limits re: significance re: inequality |

**AUTHORITY**

**Individual author:**

• Associated with a reputable organisation?

• Professional qualifications or considerable experience?

• Produced/published other work (grey/black) in the field?

• Recognised expert, identified in other sources?

• Cited by others? (use Google Scholar as a quick check)

• Higher degree student under “expert” supervision?

**Organisation or group**:

• Is the organisation reputable? (e.g. W.H.O)

• Is the organisation an authority in the field?

**In all cases:**

• Does the item have a detailed reference list or bibliography?

**ACCURACY**

• Does the item have a clearly stated aim or brief?

• Is so, is this met?

• Does it have a stated methodology?

• If so, is it adhered to?

• Has it been peer-reviewed?

• Has it been edited by a reputable authority?

• Supported by authoritative, documented references or credible sources?

• Is it representative of work in the field?

• If No, is it a valid counterbalance?

• Is any data collection explicit and appropriate for the research?

• If item is secondary material (e.g. a policy brief of a technical report) refer to the original. Is it an accurate, unbiased interpretation or analysis?

**COVERAGE**

All items have parameters which define their content coverage. These limits might mean that a work refers to a particular population group, or that it excluded certain types of publication. A report could be designed to answer a particular question, or be based on statistics from a particular survey.

• Are any limits clearly stated?

**OBJECTIVITY**

It is important to identify bias, particularly if it is unstated or unacknowledged.

• Opinion, expert or otherwise, is still opinion: is the author’s standpoint clear?

• Does the work seem to be balanced in presentation?

**DATE**

For the item to inform your research, it needs to have a date that confirms relevance

• Does the item have a clearly stated date related to content? No easily discernible date is a strong concern.

• If no date is given, but can be closely ascertained, is there a valid reason for its absence?

• Check the bibliography: have key contemporary material been included

**SIGNIFICANCE**

This is a value judgment of the item, in the context of the relevant research area

• Is the item meaningful? (this incorporates feasibility, utility and relevance)

• Does it add context?

• Does it enrich or add something unique to the research?

• Does it strengthen or refute a current position?

• Would the research area be lesser without it?

• Is it integral, representative, typical?

• Does it have impact? (in the sense of influencing the work or behaviour of others)

**Quality assessment of included systematic reviews**

Reviewer Barnes_(for all)

Author____Ahmed 2004__

|  | Yes | No | Unclear | Not applicable |
| --- | --- | --- | --- | --- |
| 1. Is the review question clearly and explicitly stated? | x | □ | □ | □ |
| 1. Were the inclusion criteria appropriate for the review question? | x | □ | □ | □ |
| 1. Was the search strategy appropriate? | X  though | □ | □ | □ |
| 1. Were the sources and resources used to search for studies adequate? | x | □ | □ | □ |
| 1. Were the criteria for appraising studies appropriate? | □ | □ | x | □ |
| 1. Was critical appraisal conducted by two or more reviewers independently? | □ | □ | x | □ |
| 1. Were there methods to minimize errors in data extraction? | x | □ | □ | □ |
| 1. Were the methods used to combine studies appropriate? | □ | □ | x | □ |
| 1. Was the likelihood of publication bias assessed? | □ | □ | x | □ |
| 1. Were recommendations for policy and/or practice supported by the reported data? | x | □ | □ | □ |
| 1. Were the specific directives for new research appropriate? | x | □ | □ | □ |

Author____Akarsu et al 2019

|  | Yes | No | Unclear | Not applicable |
| --- | --- | --- | --- | --- |
| 1. Is the review question clearly and explicitly stated? | x | □ | □ | □ |
| 1. Were the inclusion criteria appropriate for the review question? | x | □ | □ | □ |
| 1. Was the search strategy appropriate? | x | □ | □ | □ |
| 1. Were the sources and resources used to search for studies adequate? | x | □ | □ | □ |
| 1. Were the criteria for appraising studies appropriate? | x | □ | □ | □ |
| 1. Was critical appraisal conducted by two or more reviewers independently? | x | □ | □ | □ |
| 1. Were there methods to minimize errors in data extraction? | x | □ | □ | □ |
| 1. Were the methods used to combine studies appropriate? | □ | □ | X (for barriers/facilitators section?) | □ |
| 1. Was the likelihood of publication bias assessed? | □ | □ | x | □ |
| 1. Were recommendations for policy and/or practice supported by the reported data? | x | □ | □ | □ |
| 1. Were the specific directives for new research appropriate? | x | □ | □ | □ |

Author____Buczak-Stec et al 2023

|  | Yes | No | Unclear | Not applicable |
| --- | --- | --- | --- | --- |
| 1. Is the review question clearly and explicitly stated? | x | □ | □ | □ |
| 1. Were the inclusion criteria appropriate for the review question? | x | □ | □ | □ |
| 1. Was the search strategy appropriate? | x | □ | □ | □ |
| 1. Were the sources and resources used to search for studies adequate? | x | □ | □ | □ |
| 1. Were the criteria for appraising studies appropriate? | x | □ | □ | □ |
| 1. Was critical appraisal conducted by two or more reviewers independently? | x | □ | □ | □ |
| 1. Were there methods to minimize errors in data extraction? | □ | □ | x | □ |
| 1. Were the methods used to combine studies appropriate? | x | □ | □ | □ |
| 1. Was the likelihood of publication bias assessed? | □ | □ | x | □ |
| 1. Were recommendations for policy and/or practice supported by the reported data? | x | □ | □ | □ |
| 1. Were the specific directives for new research appropriate? | x | □ | □ | □ |

Author Chacko et al 2022

|  | Yes | No | Unclear | Not applicable |
| --- | --- | --- | --- | --- |
| 1. Is the review question clearly and explicitly stated? | x | □ | □ | □ |
| 1. Were the inclusion criteria appropriate for the review question? | x | □ | □ | □ |
| 1. Was the search strategy appropriate? | x | □ | □ | □ |
| 1. Were the sources and resources used to search for studies adequate? | x | □ | □ | □ |
| 1. Were the criteria for appraising studies appropriate? | x | □ | □ | □ |
| 1. Was critical appraisal conducted by two or more reviewers independently? | x | □ | □ | □ |
| 1. Were there methods to minimize errors in data extraction? | x | □ | □ | □ |
| 1. Were the methods used to combine studies appropriate? | x | □ | □ | □ |
| 1. Was the likelihood of publication bias assessed? | x | □ | □ | □ |
| 1. Were recommendations for policy and/or practice supported by the reported data? | x | □ | □ | □ |
| 1. Were the specific directives for new research appropriate? | x | □ | □ | □ |

Author____Connolly et al 2012

|  | Yes | No | Unclear | Not applicable |
| --- | --- | --- | --- | --- |
| 1. Is the review question clearly and explicitly stated? | x | □ | □ | □ |
| 1. Were the inclusion criteria appropriate for the review question? | x | □ | □ | □ |
| 1. Was the search strategy appropriate? | □ | □ | x | □ |
| 1. Were the sources and resources used to search for studies adequate? | x | □ | □ | □ |
| 1. Were the criteria for appraising studies appropriate? | x | □ | □ | □ |
| 1. Was critical appraisal conducted by two or more reviewers independently? | x | □ | □ | □ |
| 1. Were there methods to minimize errors in data extraction? | □ | □ | x | □ |
| 1. Were the methods used to combine studies appropriate? | □ | □ | x | □ |
| 1. Was the likelihood of publication bias assessed? | □ | □ | x | □ |
| 1. Were recommendations for policy and/or practice supported by the reported data? | x | □ | □ | □ |
| 1. Were the specific directives for new research appropriate? | x | □ | □ | □ |

Author____Duran-Kiraç etal 2021

|  | Yes | No | Unclear | Not applicable |
| --- | --- | --- | --- | --- |
| 1. Is the review question clearly and explicitly stated? | x | □ | □ | □ |
| 1. Were the inclusion criteria appropriate for the review question? | x | □ | □ | □ |
| 1. Was the search strategy appropriate? | □ | □ | x | □ |
| 1. Were the sources and resources used to search for studies adequate? | □ | □ | x | □ |
| 1. Were the criteria for appraising studies appropriate? | x | □ | □ | □ |
| 1. Was critical appraisal conducted by two or more reviewers independently? | x | □ | □ | □ |
| 1. Were there methods to minimize errors in data extraction? | x | □ | □ | □ |
| 1. Were the methods used to combine studies appropriate? | x | □ | □ | □ |
| 1. Was the likelihood of publication bias assessed? | □ | □ | x | □ |
| 1. Were recommendations for policy and/or practice supported by the reported data? | x | □ | □ | □ |
| 1. Were the specific directives for new research appropriate? | x | □ | □ | □ |

Author____Gilmore-Bykovskyi et al 2018

|  | Yes | No | Unclear | Not applicable |
| --- | --- | --- | --- | --- |
| 1. Is the review question clearly and explicitly stated? | x | □ | □ | □ |
| 1. Were the inclusion criteria appropriate for the review question? | x | □ | □ | □ |
| 1. Was the search strategy appropriate? | x | □ | □ | □ |
| 1. Were the sources and resources used to search for studies adequate? | x | □ | □ | □ |
| 1. Were the criteria for appraising studies appropriate? | x | □ | □ | □ |
| 1. Was critical appraisal conducted by two or more reviewers independently? | □ | □ | x | □ |
| 1. Were there methods to minimize errors in data extraction? | x | □ | □ | □ |
| 1. Were the methods used to combine studies appropriate? | x | □ | □ | □ |
| 1. Was the likelihood of publication bias assessed? | □ | □ | x | □ |
| 1. Were recommendations for policy and/or practice supported by the reported data? | x | □ | □ | □ |
| 1. Were the specific directives for new research appropriate? | x | □ | □ | □ |

Author____Greenwood et al 2015

|  | Yes | No | Unclear | Not applicable |
| --- | --- | --- | --- | --- |
| 1. Is the review question clearly and explicitly stated? | x | □ | □ | □ |
| 1. Were the inclusion criteria appropriate for the review question? | x | □ | □ | □ |
| 1. Was the search strategy appropriate? | x | □ | □ | □ |
| 1. Were the sources and resources used to search for studies adequate? | x | □ | □ | □ |
| 1. Were the criteria for appraising studies appropriate? | x | □ | □ | □ |
| 1. Was critical appraisal conducted by two or more reviewers independently? | x | □ | □ | □ |
| 1. Were there methods to minimize errors in data extraction? | □ | □ | x | □ |
| 1. Were the methods used to combine studies appropriate? | x | □ | □ | □ |
| 1. Was the likelihood of publication bias assessed? | □ | □ | x | □ |
| 1. Were recommendations for policy and/or practice supported by the reported data? | x | □ | □ | □ |
| 1. Were the specific directives for new research appropriate? | x | □ | □ | □ |

Author____Johl et al 2016

|  | Yes | No | Unclear | Not applicable |
| --- | --- | --- | --- | --- |
| 1. Is the review question clearly and explicitly stated? | x | □ | □ | □ |
| 1. Were the inclusion criteria appropriate for the review question? | x | □ | □ | □ |
| 1. Was the search strategy appropriate? | x | □ | □ | □ |
| 1. Were the sources and resources used to search for studies adequate? | x | □ | □ | □ |
| 1. Were the criteria for appraising studies appropriate? | □ | □ | x | □ |
| 1. Was critical appraisal conducted by two or more reviewers independently? | □ | □ | x | □ |
| 1. Were there methods to minimize errors in data extraction? | □ | □ | x | □ |
| 1. Were the methods used to combine studies appropriate? | x | □ | □ | □ |
| 1. Was the likelihood of publication bias assessed? | □ | □ | x | □ |
| 1. Were recommendations for policy and/or practice supported by the reported data? | x | □ | □ | □ |
| 1. Were the specific directives for new research appropriate? | x | □ | □ | □ |

Author____ Knipping et al 2023

|  | Yes | No | Unclear | Not applicable |
| --- | --- | --- | --- | --- |
| 1. Is the review question clearly and explicitly stated? | x | □ | □ | □ |
| 1. Were the inclusion criteria appropriate for the review question? | x | □ | □ | □ |
| 1. Was the search strategy appropriate? | x | □ | □ | □ |
| 1. Were the sources and resources used to search for studies adequate? | x | □ | □ | □ |
| 1. Were the criteria for appraising studies appropriate? | □ | □ | x | □ |
| 1. Was critical appraisal conducted by two or more reviewers independently? | □ | □ | x | □ |
| 1. Were there methods to minimize errors in data extraction? | □ | □ | x | □ |
| 1. Were the methods used to combine studies appropriate? | x | □ | □ | □ |
| 1. Was the likelihood of publication bias assessed? | □ | □ | x | □ |
| 1. Were recommendations for policy and/or practice supported by the reported data? | x | □ | □ | □ |
| 1. Were the specific directives for new research appropriate? | x | □ | □ | □ |

Author____ Kokorelias et al 2023

|  | Yes | No | Unclear | Not applicable |
| --- | --- | --- | --- | --- |
| 1. Is the review question clearly and explicitly stated? | x | □ | □ | □ |
| 1. Were the inclusion criteria appropriate for the review question? | x | □ | □ | □ |
| 1. Was the search strategy appropriate? | x | □ | □ | □ |
| 1. Were the sources and resources used to search for studies adequate? | x | □ | □ | □ |
| 1. Were the criteria for appraising studies appropriate? | □ | □ | x | □ |
| 1. Was critical appraisal conducted by two or more reviewers independently? | □ | □ | X | □ |
| 1. Were there methods to minimize errors in data extraction? | □ | □ | x | □ |
| 1. Were the methods used to combine studies appropriate? | x | □ | □ | □ |
| 1. Was the likelihood of publication bias assessed? | □ | □ | X | □ |
| 1. Were recommendations for policy and/or practice supported by the reported data? | x | □ | □ | □ |
| 1. Were the specific directives for new research appropriate? | x | □ | □ | □ |

Author____ Lillekroken et al 2023

|  | Yes | No | Unclear | Not applicable |
| --- | --- | --- | --- | --- |
| 1. Is the review question clearly and explicitly stated? | x | □ | □ | □ |
| 1. Were the inclusion criteria appropriate for the review question? | x | □ | □ | □ |
| 1. Was the search strategy appropriate? | x | □ | □ | □ |
| 1. Were the sources and resources used to search for studies adequate? | x | □ | □ | □ |
| 1. Were the criteria for appraising studies appropriate? | x | □ | □ | □ |
| 1. Was critical appraisal conducted by two or more reviewers independently? | x | □ | □ | □ |
| 1. Were there methods to minimize errors in data extraction? | □ | □ | x | □ |
| 1. Were the methods used to combine studies appropriate? | □ | □ | x | □ |
| 1. Was the likelihood of publication bias assessed? | □ | □ | x | □ |
| 1. Were recommendations for policy and/or practice supported by the reported data? | x | □ | □ | □ |
| 1. Were the specific directives for new research appropriate? | x | □ | □ | □ |

Author____ Lorenz et al 2021

|  | Yes | No | Unclear | Not applicable |
| --- | --- | --- | --- | --- |
| 1. Is the review question clearly and explicitly stated? | x | □ | □ | □ |
| 1. Were the inclusion criteria appropriate for the review question? | x | □ | □ | □ |
| 1. Was the search strategy appropriate? | x | □ | □ | □ |
| 1. Were the sources and resources used to search for studies adequate? | x | □ | □ | □ |
| 1. Were the criteria for appraising studies appropriate? | □ | □ | x | □ |
| 1. Was critical appraisal conducted by two or more reviewers independently? | □ | □ | x | □ |
| 1. Were there methods to minimize errors in data extraction? | x | □ | □ | □ |
| 1. Were the methods used to combine studies appropriate? | x | □ | □ | □ |
| 1. Was the likelihood of publication bias assessed? | □ | □ | x | □ |
| 1. Were recommendations for policy and/or practice supported by the reported data? | x | □ | □ | □ |
| 1. Were the specific directives for new research appropriate? | x | □ | □ | □ |

Author____ Lucero et al 2018

|  | Yes | No | Unclear | Not applicable |
| --- | --- | --- | --- | --- |
| 1. Is the review question clearly and explicitly stated? | x | □ | □ | □ |
| 1. Were the inclusion criteria appropriate for the review question? | x | □ | □ | □ |
| 1. Was the search strategy appropriate? | x | □ | □ | □ |
| 1. Were the sources and resources used to search for studies adequate? | x | □ | □ | □ |
| 1. Were the criteria for appraising studies appropriate? | x | □ | □ | □ |
| 1. Was critical appraisal conducted by two or more reviewers independently? | x | □ | □ | □ |
| 1. Were there methods to minimize errors in data extraction? | x | □ | □ | □ |
| 1. Were the methods used to combine studies appropriate? | □ | □ | x | □ |
| 1. Was the likelihood of publication bias assessed? | □ | □ | x | □ |
| 1. Were recommendations for policy and/or practice supported by the reported data? | x | □ | □ | □ |
| 1. Were the specific directives for new research appropriate? | x | □ | □ | □ |

Author____ Murong et al 2023

|  | Yes | No | Unclear | Not applicable |
| --- | --- | --- | --- | --- |
| 1. Is the review question clearly and explicitly stated? | x | □ | □ | □ |
| 1. Were the inclusion criteria appropriate for the review question? | x | □ | □ | □ |
| 1. Was the search strategy appropriate? | x | □ | □ | □ |
| 1. Were the sources and resources used to search for studies adequate? | x | □ | □ | □ |
| 1. Were the criteria for appraising studies appropriate? | □ | □ | x | □ |
| 1. Was critical appraisal conducted by two or more reviewers independently? | □ | □ | x | □ |
| 1. Were there methods to minimize errors in data extraction? | □ | □ | x | □ |
| 1. Were the methods used to combine studies appropriate? | □ | □ | x | □ |
| 1. Was the likelihood of publication bias assessed? | □ | □ | x | □ |
| 1. Were recommendations for policy and/or practice supported by the reported data? | x | □ | □ | □ |
| 1. Were the specific directives for new research appropriate? | x | □ | □ | □ |

Author____ Napoles et al

|  | Yes | No | Unclear | Not applicable |
| --- | --- | --- | --- | --- |
| 1. Is the review question clearly and explicitly stated? | x | □ | □ | □ |
| 1. Were the inclusion criteria appropriate for the review question? | x | □ | □ | □ |
| 1. Was the search strategy appropriate? | □ | □ | x | □ |
| 1. Were the sources and resources used to search for studies adequate? | x | □ | □ | □ |
| 1. Were the criteria for appraising studies appropriate? | □ | □ | x | □ |
| 1. Was critical appraisal conducted by two or more reviewers independently? | □ | □ | x | □ |
| 1. Were there methods to minimize errors in data extraction? | □ | □ | x | □ |
| 1. Were the methods used to combine studies appropriate? | x | □ | □ | □ |
| 1. Was the likelihood of publication bias assessed? | □ | □ | x | □ |
| 1. Were recommendations for policy and/or practice supported by the reported data? | x | □ | □ | □ |
| 1. Were the specific directives for new research appropriate? | x | □ | □ | □ |

Author____ San Juan et al 2021

|  | Yes | No | Unclear | Not applicable |
| --- | --- | --- | --- | --- |
| 1. Is the review question clearly and explicitly stated? | x | □ | □ | □ |
| 1. Were the inclusion criteria appropriate for the review question? | x | □ | □ | □ |
| 1. Was the search strategy appropriate? | x | □ | □ | □ |
| 1. Were the sources and resources used to search for studies adequate? | x | □ | □ | □ |
| 1. Were the criteria for appraising studies appropriate? | x | □ | □ | □ |
| 1. Was critical appraisal conducted by two or more reviewers independently? | x | □ | □ | □ |
| 1. Were there methods to minimize errors in data extraction? | x | □ | □ | □ |
| 1. Were the methods used to combine studies appropriate? | x | □ | □ | □ |
| 1. Was the likelihood of publication bias assessed? | x | □ | □ | □ |
| 1. Were recommendations for policy and/or practice supported by the reported data? | x | □ | □ | □ |
| 1. Were the specific directives for new research appropriate? | x | □ | □ | □ |

Author____ Shrestha 2023

|  | Yes | No | Unclear | Not applicable |
| --- | --- | --- | --- | --- |
| 1. Is the review question clearly and explicitly stated? | x | □ | □ | □ |
| 1. Were the inclusion criteria appropriate for the review question? | x | □ | □ | □ |
| 1. Was the search strategy appropriate? | x | □ | □ | □ |
| 1. Were the sources and resources used to search for studies adequate? | x | □ | □ | □ |
| 1. Were the criteria for appraising studies appropriate? | x | □ | □ | □ |
| 1. Was critical appraisal conducted by two or more reviewers independently? | x | □ | □ | □ |
| 1. Were there methods to minimize errors in data extraction? | x | □ | □ | □ |
| 1. Were the methods used to combine studies appropriate? | x | □ | □ | □ |
| 1. Was the likelihood of publication bias assessed? | □ | □ | x | □ |
| 1. Were recommendations for policy and/or practice supported by the reported data? | x | □ | □ | □ |
| 1. Were the specific directives for new research appropriate? | x | □ | □ | □ |

Author____ Thompson et al 2020

|  | Yes | No | Unclear | Not applicable |
| --- | --- | --- | --- | --- |
| 1. Is the review question clearly and explicitly stated? | x | □ | □ | □ |
| 1. Were the inclusion criteria appropriate for the review question? | x | □ | □ | □ |
| 1. Was the search strategy appropriate? | x | □ | □ | □ |
| 1. Were the sources and resources used to search for studies adequate? | x | □ | □ | □ |
| 1. Were the criteria for appraising studies appropriate? | x | □ | □ | □ |
| 1. Was critical appraisal conducted by two or more reviewers independently? | x | □ | □ | □ |
| 1. Were there methods to minimize errors in data extraction? | x | □ | □ | □ |
| 1. Were the methods used to combine studies appropriate? | x | □ | □ | □ |
| 1. Was the likelihood of publication bias assessed? | x | □ | □ | □ |
| 1. Were recommendations for policy and/or practice supported by the reported data? | x | □ | □ | □ |
| 1. Were the specific directives for new research appropriate? | x | □ | □ | □ |

Author____Waligora et al 2018

|  | Yes | No | Unclear | Not applicable |
| --- | --- | --- | --- | --- |
| 1. Is the review question clearly and explicitly stated? | x | □ | □ | □ |
| 1. Were the inclusion criteria appropriate for the review question? | x | □ | □ | □ |
| 1. Was the search strategy appropriate? | x | □ | □ | □ |
| 1. Were the sources and resources used to search for studies adequate? | x | □ | □ | □ |
| 1. Were the criteria for appraising studies appropriate? | x | □ | □ | □ |
| 1. Was critical appraisal conducted by two or more reviewers independently? | x | □ | □ | □ |
| 1. Were there methods to minimize errors in data extraction? | □ | □ | x | □ |
| 1. Were the methods used to combine studies appropriate? | x | □ | □ | □ |
| 1. Was the likelihood of publication bias assessed? | □ | □ | x | □ |
| 1. Were recommendations for policy and/or practice supported by the reported data? | x | □ | □ | □ |
| 1. Were the specific directives for new research appropriate? | x | □ | □ | □ |

Author____ Zhang et al

|  | Yes | No | Unclear | Not applicable |
| --- | --- | --- | --- | --- |
| 1. Is the review question clearly and explicitly stated? | x | □ | □ | □ |
| 1. Were the inclusion criteria appropriate for the review question? | x | □ | □ | □ |
| 1. Was the search strategy appropriate? | x | □ | □ | □ |
| 1. Were the sources and resources used to search for studies adequate? | x | □ | □ | □ |
| 1. Were the criteria for appraising studies appropriate? | x | □ | □ | □ |
| 1. Was critical appraisal conducted by two or more reviewers independently? | x | □ | □ | □ |
| 1. Were there methods to minimize errors in data extraction? | x | □ | □ | □ |
| 1. Were the methods used to combine studies appropriate? | x | □ | □ | □ |
| 1. Was the likelihood of publication bias assessed? | □ | □ | x | □ |
| 1. Were recommendations for policy and/or practice supported by the reported data? | x | □ | □ | □ |
| 1. Were the specific directives for new research appropriate? | x | □ | □ | □ |

Author____

|  | Yes | No | Unclear | Not applicable |
| --- | --- | --- | --- | --- |
| 1. Is the review question clearly and explicitly stated? | x | □ | □ | □ |
| 1. Were the inclusion criteria appropriate for the review question? | x | □ | □ | □ |
| 1. Was the search strategy appropriate? | □ | □ | x | □ |
| 1. Were the sources and resources used to search for studies adequate? | x | □ | □ | □ |
| 1. Were the criteria for appraising studies appropriate? | □ | □ | x | □ |
| 1. Was critical appraisal conducted by two or more reviewers independently? | □ | □ | x | □ |
| 1. Were there methods to minimize errors in data extraction? | □ | □ | x | □ |
| 1. Were the methods used to combine studies appropriate? | x | □ | □ | □ |
| 1. Was the likelihood of publication bias assessed? | □ | □ | x | □ |
| 1. Were recommendations for policy and/or practice supported by the reported data? | x | □ | □ | □ |
| 1. Were the specific directives for new research appropriate? | x | □ | □ | □ |

Author____ Zarzycki, et al. 2023

|  | Yes | No | Unclear | Not applicable |
| --- | --- | --- | --- | --- |
| 1. Is the review question clearly and explicitly stated? | x | □ | □ | □ |
| 1. Were the inclusion criteria appropriate for the review question? | x | □ | □ | □ |
| 1. Was the search strategy appropriate? | x | □ | □ | □ |
| 1. Were the sources and resources used to search for studies adequate? | x | □ | □ | □ |
| 1. Were the criteria for appraising studies appropriate? | x | □ | □ | □ |
| 1. Was critical appraisal conducted by two or more reviewers independently? | x | □ | □ | □ |
| 1. Were there methods to minimize errors in data extraction? | x | □ | □ | □ |
| 1. Were the methods used to combine studies appropriate? | x | □ | □ | □ |
| 1. Was the likelihood of publication bias assessed? | □ | □ | x | □ |
| 1. Were recommendations for policy and/or practice supported by the reported data? | x | □ | □ | □ |
| 1. Were the specific directives for new research appropriate? | x | □ | □ | □ |

Author____ Ireson et al 2016

|  | Yes | No | Unclear | Not applicable |
| --- | --- | --- | --- | --- |
| 1. Is the review question clearly and explicitly stated? | x | □ | □ | □ |
| 1. Were the inclusion criteria appropriate for the review question? | x | □ | □ | □ |
| 1. Was the search strategy appropriate? | x | □ | □ | □ |
| 1. Were the sources and resources used to search for studies adequate? | x | □ | □ | □ |
| 1. Were the criteria for appraising studies appropriate? | □ | □ | x | □ |
| 1. Was critical appraisal conducted by two or more reviewers independently? | □ | □ | x | □ |
| 1. Were there methods to minimize errors in data extraction? | x | □ | □ | □ |
| 1. Were the methods used to combine studies appropriate? | x | □ | □ | □ |
| 1. Was the likelihood of publication bias assessed? | □ | □ | x | □ |
| 1. Were recommendations for policy and/or practice supported by the reported data? | x | □ | □ | □ |
| 1. Were the specific directives for new research appropriate? | x | □ | □ | □ |

Author____ Francis and Hanna 2022

|  | Yes | No | Unclear | Not applicable |
| --- | --- | --- | --- | --- |
| 1. Is the review question clearly and explicitly stated? | x | □ | □ | □ |
| 1. Were the inclusion criteria appropriate for the review question? | x | □ | □ | □ |
| 1. Was the search strategy appropriate? | x | □ | □ | □ |
| 1. Were the sources and resources used to search for studies adequate? | x | □ | □ | □ |
| 1. Were the criteria for appraising studies appropriate? | □ | □ | x | □ |
| 1. Was critical appraisal conducted by two or more reviewers independently? | □ | □ | x | □ |
| 1. Were there methods to minimize errors in data extraction? | □ | □ | x | □ |
| 1. Were the methods used to combine studies appropriate? | x | □ | □ | □ |
| 1. Was the likelihood of publication bias assessed? | □ | □ | x | □ |
| 1. Were recommendations for policy and/or practice supported by the reported data? | x | □ | □ | □ |
| 1. Were the specific directives for new research appropriate? | x | □ | □ | □ |

Author____ Stenberg and Hejlem 2023

|  | Yes | No | Unclear | Not applicable |
| --- | --- | --- | --- | --- |
| 1. Is the review question clearly and explicitly stated? | x | □ | □ | □ |
| 1. Were the inclusion criteria appropriate for the review question? | x | □ | □ | □ |
| 1. Was the search strategy appropriate? | x | □ | □ | □ |
| 1. Were the sources and resources used to search for studies adequate? | x | □ | □ | □ |
| 1. Were the criteria for appraising studies appropriate? | x | □ | □ | □ |
| 1. Was critical appraisal conducted by two or more reviewers independently? | x | □ | □ | □ |
| 1. Were there methods to minimize errors in data extraction? | x | □ | □ | □ |
| 1. Were the methods used to combine studies appropriate? | x | □ | □ | □ |
| 1. Was the likelihood of publication bias assessed? | □ | □ | x | □ |
| 1. Were recommendations for policy and/or practice supported by the reported data? | x | □ | □ | □ |
| 1. Were the specific directives for new research appropriate? | x | □ | □ | □ |

**Quality assessment of qualitative papers**

| **First author, date** | **1** | **2** | **3** | **4** | **5** | **6** | **7** | **8** | **9** | **10** |
| --- | --- | --- | --- | --- | --- | --- | --- | --- | --- | --- |
| Moss et al | yes | yes | yes | Not clear why CCG used | yes | no | unclear | yes | yes | Some relevance to understanding needs of and inequalities experienced by minoritised unpaid carers particularly in relation to palliative care. |

For each, Yes, Not clear or No

1. Was there a clear statement of the aims of the research?
2. Was a qualitative methodology appropriate?
3. Was the research design appropriate to address the aims of the research?
4. Was the recruitment strategy appropriate to the aims of the research?
5. Was the data collected in a way that addressed the research issue?
6. Has the relationship between researcher and participants been adequately considered?
7. Have ethical issues been taken into consideration?
8. Was the data analysis sufficiently rigorous?
9. Is there a clear statement of findings?
10. How valuable is the research?
